# A simpler linear-time algorithm for the common refinement of rooted phylogenetic trees on a common leaf set

**DOI:** 10.1186/s13015-021-00202-8

**Published:** 2021-12-06

**Authors:** David Schaller, Marc Hellmuth, Peter F. Stadler

**Affiliations:** 1 Bioinformatics Group, Department of Computer Science, and Interdisciplinary Center for Bioinformatics, Universität Leipzig, Härtelstraße 16–18, 04107 Leipzig, Germany; 2 Department of Mathematics, Faculty of Science, Stockholm University, SE-10691 Stockholm, Sweden; 3 Competence Center for Scalable Data Services and Solutions Dresden/Leipzig, Interdisciplinary Center for Bioinformatics, German Centre for Integrative Biodiversity Research (iDiv), and Leipzig Research Center for Civilization Diseases, Universität Leipzig, Augustusplatz 12, 04107 Leipzig, Germany; 4 Max Planck Institute for Mathematics in the Sciences, Inselstraße 22, 04109 Leipzig, Germany; 5 Department of Theoretical Chemistry, University of Vienna, Währinger Straße 17, 1090 Vienna, Austria; 6 Facultad de Ciencias, Universidad National de Colombia, Sede Bogotá, Ciudad Universitaria, Bogotá, 111321 D.C Colombia; 7 Santa Fe Institute, 1399 Hyde Park Rd., Santa Fe, NM 87501 USA

**Keywords:** Mathematical phylogenetics, Rooted trees, Compatibility of rooted trees

## Abstract

**Background:**

The supertree problem, i.e., the task of finding a common refinement of a set of rooted trees is an important topic in mathematical phylogenetics. The special case of a common leaf set *L* is known to be solvable in linear time. Existing approaches refine one input tree using information of the others and then test whether the results are isomorphic.

**Results:**

An *O*(*k*|*L*|) algorithm, LinCR, for constructing the common refinement *T* of *k* input trees with a common leaf set *L* is proposed that explicitly computes the parent function of *T* in a bottom-up approach.

**Conclusion:**

LinCR is simpler to implement than other asymptotically optimal algorithms for the problem and outperforms the alternatives in empirical comparisons.

**Availability:**

An implementation of LinCR in Python is freely available at https://github.com/david-schaller/tralda.

## Introduction

Given a collection of rooted phylogenetic trees $T_1$, $T_2$, ...$T_k$, the supertree problem in phylogenetics consists in determining whether there is a common tree *T* that “displays” all input trees $T_i$, $1\le i\le k$, and if so, a supertree *T* is to be constructed [1, 2]. In its most general form, the leaf sets $L(T_i)$, representing the taxonomic units (taxa), may differ, and the supertree *T* has the leaf set $L(T)=\bigcup _{i=1}^k L(T_i)$. Writing $n{:}{=}|L(T)|$, $N{:}{=}\sum _{i=1}^k |L(T_i)|$, and $R{:}{=}\sum _{i=1}^k |L(T_i)|^2$, this problem is solved by the algorithm of Aho et al. [3], which is commonly called BUILD in the the phylogenetic literature [4], in *O*(*Nn*) time for binary trees and *O*(*Rn*) time in general.

An $O(N^2)$ algorithm to compute all binary trees compatible with the input is described in [5]. Using sophisticated data structures, the effort for computing a single supertree was reduced to $O(\min (N \sqrt{n},N+n^2\log n))$ for binary trees and $(R\log ^2 R)$ for arbitrary input trees [6]. Recently, an $O(N \log ^2 N)$ algorithm has become available for the compatibility problem for general trees [7]. The compatibility problem for nested taxa in addition assigns labels to inner vertices and can also be solved in $O(N \log ^2 N)$ [8].

Here we consider the special case that the input trees share the same leaf set $L(T_1)=L(T_2)=\dots =L(T_k)=L(T)=L$, and thus $N=kn$ and $R=kn^2$. While the general supertree problem arises naturally when attempting to reconcile phylogenetic trees produced in independent studies, the special case appears in particular when incompletely resolved trees are produced with different methods. In a recent work, we have shown that such trees can be inferred e.g. as the least resolved trees from best match data [9, 10] and from information of horizontal gene transfer [11, 12]. Denoting with ${\mathcal {H}}(T)$ the set of “clusters” in *T*, we recently showed that the latter type of data can be explained by a common evolutionary scenario if and only if (1) both the best match and the horizontal transfer data can be explained by least resolved trees $T_1$ and $T_2$, respectively, and (2) the union ${\mathcal {H}}(T_1)\cup {\mathcal {H}}(T_2)$ is again a hierarchy. In this context it is of practical interest whether the latter property can be tested efficiently, and whether the common refinement *T* satisfying ${\mathcal {H}}(T)={\mathcal {H}}(T_1)\cup {\mathcal {H}}(T_2)$ [13] can be constructed efficiently in the positive case.

Several linear time, i.e., *O*(|*L*|) time, algorithms for the common refinement of two input trees $T_1$ and $T_2$ with a common leaf set have become available. The INSERT algorithm [14], which makes use of ideas from [15], inserts the clusters of $T_2$ into $T_1$ and *vice versa* and then uses a linear-time algorithm to check whether the two edited trees are isomorphic [16]. Assuming that the input trees are already known to be compatible, ${\texttt {Merge\_Trees}}$ [17, 18] can also be applied to insert the clusters of one tree into the other. For both of these methods, an overall linear-time algorithm for the common refinement of *k* input trees is then obtained by iteratively computing the common refinement of the input tree $T_j$ and the common refinement of first $j-1$ trees, resulting in a total effort of *O*(*k*|*L*|).

Here we describe an alternative algorithm that constructs in a single step a candidate refinement *T* of all *k* input trees. This is achieved by computing the parent-function of the potential refinement *T* in a bottom-up fashion. As we shall see, the algorithm is easy to implement and does not require elaborate data structures. The existence of a common refinement is then verified by checking that the parent function defines a tree *T* and, if so, that *T* displays each of the input trees $T_j$. This test is also much simpler to implement than the isomorphism test for rooted trees [16].

## Theory

### Notation and preliminaries

Let *T* be a rooted tree. We write *V*(*T*) for its vertex set, *E*(*T*) for is edge set, $L(T)\subseteq V(T)$ for its leaf set, $V^0(T){:}{=}V(T)\setminus L(T)$ for the set of inner vertices and $\rho \in V^0(T)$ for its root. An edge $e=\{u,v\}\in E(T)$ is an *inner* edge if $u,v\in V^0(T)$. The ancestor partial order $\preceq _T$ on *V*(*T*) is defined by $x\preceq _T y$ whenever *y* lies along the unique path connecting *x* and the root $\rho$. If $x\preceq _T y$ and $x\ne y$, we write $x \prec _T y$. For $v\in V(T)$, we set ${\text {child}}_T(v){:}{=}\{u\mid \{v,u\}\in E(T),\, u\prec _T v\}$. If $u\in {\text {child}}_T(v)$, then *v* is the unique parent of *u*. In this case, we write $v={\text {parent}}_T(u)$. All trees *T* considered in this contribution are *phylogenetic*, i.e., they satisfy $|{\text {child}}_T(v)|\ge 2$ for all $v\in V^0(T)$.

We denote by *T*(*u*) the subtree of *T* rooted in *u* and write *L*(*T*(*u*)) for its leaf set. The *last common ancestor* of a vertex set $W\subseteq V(T)$ is the unique $\preceq _T$-minimal vertex ${\text {lca}}_T(W)\in V(T)$ satisfying $w\preceq _T{\text {lca}}_T(W)$ for all $w\in W$. For brevity, we write ${\text {lca}}_T(x,y){:}{=}{\text {lca}}_T(\{x,y\})$. Furthermore, we will sometimes write $vu\in E(T)$ as a shorthand for “$\{u,v\}\in E(T)$ with $u\prec _T v$.”

A hierarchy on *L* is set system ${\mathcal {H}}\subseteq 2^L$ such that (i) $L\in {\mathcal {H}}$, (ii) $A\cap B\in \{A,B,\emptyset \}$ for all $A,B\in {\mathcal {H}}$, and (iii) $\{x\}\in {\mathcal {H}}$ for all $x\in L$. There is a well-known bijection between rooted phylogenetic trees *T* with leaf set *L* and hierarchies on *L*, see e.g. [4, Thm. 3.5.2]. It is given by ${\mathcal {H}}(T) {:}{=}\{ L(T(u)) \mid u\in V(T) \}$; conversely, the tree $T_{{\mathcal {H}}}$ corresponding to a hierarchy ${\mathcal {H}}$ is the Hasse diagram w.r.t. set inclusion. Thus, if $v={\text {lca}}_T(A)$ for some $A\subseteq L(T)$, then *L*(*T*(*v*)) is the inclusion-minimal cluster in ${\mathcal {H}}(T)$ that contains *A*, see e.g. [19]. We call the elements of ${\mathcal {H}}(T)$ *clusters* and say that two clusters *C* and $C'$ are *compatible* if $C\cap C'\in \{C,C',\emptyset \}$. Note that, by (i), the clusters of the same tree are all pairwise compatible.

A (rooted) triple is a binary tree on three leaves. We say that a tree *T* displays a triple *xy*|*z* if ${\text {lca}}_T(x,y)\prec _T{\text {lca}}_T(x,z)={\text {lca}}_T(y,z)$, or equivalently, if there is a cluster $C\in {\mathcal {H}}(T)$ such that $x,y \in C$ and $z\notin C$. The set of all triples that are displayed by *T* is denoted by *r*(*T*). A set ${\mathcal {R}}$ of triples is *consistent* if there is a tree that displays all triples in ${\mathcal {R}}$.

Let *T* and $T^*$ be phylogenetic trees with $L(T)=L(T^*)$. We say that $T^*$ is a *refinement* of *T* if *T* can be obtained from $T^*$ by contracting a subset of inner edges. Equivalently, $T^*$ is a refinement of *T* if and only if ${\mathcal {H}}(T)\subseteq {\mathcal {H}}(T^*)$. A tree *T* *displays* a tree $T'$ if $L(T')\subseteq L(T)$ and ${\mathcal {H}}(T')\subseteq \{C\cap L(T') \mid C\in {\mathcal {H}}(T) \text { and } C\cap L(T')\ne \emptyset \}$. In particular, therefore, *T* displays a tree $T'$ with $L(T')=L(T)$ if and only if ${\mathcal {H}}(T')\subseteq {\mathcal {H}}(T)$, i.e., if and only if *T* is a refinement of $T'$. The minimal *common refinement* of the trees $T_i$, $1\le i\le k$ is the tree *T* such that ${\mathcal {H}}(T)=\bigcup _{i=1}^k {\mathcal {H}}(T_i)$, provided it exists.

Thm. 3.5.2 of [4] can be rephrased in the following form:

#### Lemma 1

*Let* $T_1$, $T_2$, ..., $T_k$ *be trees with common leaf set* $L(T_i)=L$ *such that* ${\mathcal {H}}{:}{=}\bigcup _{i=1}^k{\mathcal {H}}(T_i)$ *is a hierarchy. Then there is a unique tree* *T such that* ${\mathcal {H}}(T)={\mathcal {H}}$. *Furthermore*, *T* *is the unique “least resolved” tree in the sense that contraction of any edge in T* *yields a tree* $T_e$ *with* ${\mathcal {H}}(T_e)\subsetneq {\mathcal {H}}(T)$.

#### *Proof*

By definition of ${\mathcal {H}}$ and the bijection between phylogenetic trees and hierarchies, there is a unique tree *T* such that ${\mathcal {H}}={\mathcal {H}}(T)$. Consider an inner edge $e=uv$. By construction, there is at least one tree $T_v$ such that $C{:}{=}L(T(v))\in {\mathcal {H}}(T_v)$. However, ${\mathcal {H}}(T_e)={\mathcal {H}}(T)\setminus \{C_v\}$ and thus $T_e$ does not display $T_v$. $\square$

By Thm. 1 in [20], a tree $T'$ is displayed by a tree *T* with $L(T')\subseteq L(T)$ if and only if $r(T')\subseteq r(T)$. As an immediate consequence, a common refinement of trees with a common leaf set *L* exists if and only if the union *L* of their triple sets is consistent. The latter condition can be checked using the BUILD algorithm which, in the positive case, returns a tree $\texttt {BUILD}({\mathcal {R}},L)$ that displays all triples in ${\mathcal {R}}$.

#### Lemma 2

*Suppose that* *T* *is the unique least resolved common refinement of the trees* $T_1$, $T_2$, ..., $T_k$ *with common leaf set* $L(T_i)=L$, $1\le i\le k$ *and let* ${\mathcal {R}}{:}{=}r(T_i)\cup r(T_2)\cup \dots \cup r(T_k)$. *Then* $T=\texttt {BUILD}({\mathcal {R}},L)$.

#### *Proof*

The tree ${\widehat{T}}{:}{=}\texttt {BUILD}({\mathcal {R}},L)$ is a common refinement since, by the arguments above, it displays $T_1$, $T_2$, ..., $T_k$. By Lemma 1, we therefore have ${\mathcal {H}}(T)\subseteq {\mathcal {H}}({\widehat{T}})$. Prop. 4.1 in [21] implies that ${\widehat{T}}$ is least resolved w.r.t. ${\mathcal {R}}$, i.e., every tree ${\widehat{T}}'$ obtained from ${\widehat{T}}$ by contraction of an edge no longer displays all input triples in ${\mathcal {R}}$. By Thm. 6.4.1 in [4], $T_i$ is displayed by ${\widehat{T}}'$ if and only if ${\widehat{T}}'$ displays all triples of $T_i$. Since this is not true for all input trees $T_i$, ${\widehat{T}}'$ does not display all input trees $T_i$, $1\le i\le k$. Together with ${\mathcal {H}}(T)\subseteq {\mathcal {H}}(({{\widehat{T}}}))$, this implies that $T={\widehat{T}}$. $\square$

We note that, given a set of triples *R*, “*T* is a least resolved displaying *R*” does not imply that vertex set *V*(*T*) is minimal among all such trees. It is possible in general that there is a tree $T'$ displaying a given triple set *R* with $|V(T')|<|V(\texttt {BUILD}(R, L))|$. In this case, $\texttt {BUILD}(R,L)$ does not display $T'$, see [22] for details. However, uniqueness of the least resolved tree, Lemma 1, rules out this scenario in our setting.

The algorithm BuildST [7] computes the supertree of a set ${\mathcal {T}}{:}{=}\{T_i\mid 1\le i\le k\}$ of rooted trees without first breaking down each tree to its triple set $r(T_i)$. Lemma 5 in [7] establishes that BuildST applied to a set of trees and BUILD applied to the triple set ${\mathcal {R}}{:}{=}\bigcup _{i=1}^k r(T_i)$ produce the same output for all instances. If ${\mathcal {R}}$ is consistent, BuildST computes the tree $\texttt {BUILD}({\mathcal {R}},L)$. If all input trees have the same same leaf set *L* BuildST in particular computes their common refinement. The performance analysis in [7] shows that BuildST runs in $O(k|L| \log ^2(k|L|))$ time for this special case. Linear-time algorithms for the special case of a common leaf set therefore offer a further improvement over the best known general purpose supertree algorithms.

### A bottom-up linear time algorithm

The basic idea of our approach is to construct *T* by means of a simple bottom-up approach that computes the parent function ${\text {parent}}_T:V(T)\setminus \{\rho _T\}\rightarrow V(T)\setminus L$ of a candidate tree *T* in a stepwise manner. This algorithm is based on three simple observations:

(i) If it exists, the common refinement *T* of $T_1$, $T_2$, ..., $T_k$ is uniquely defined by virtue of ${\mathcal {H}}(T)=\bigcup _{i=1}^k {\mathcal {H}}(T_i)$ (cf. Lemma 1). We will therefore identify all vertices $v_i\in V(T_i)$ with a vertex *v* in the prospective tree *T* whenever their clusters – i.e., the sets $L(T_i(v_i))$ – are the same. In this case, we have $L(T(v)){:}{=}L(T_i(v_i))$. From here on, we simply say, by a slight abuse of notation, that *v* is also a vertex of $T_i$ and write $v\in V(T_i)$.
(ii) Since ${\mathcal {H}}(T)=\bigcup _{i=1}^k {\mathcal {H}}(T_i)$, each vertex $v\in V(T)$ is also a vertex in at least one input tree $T_i$. Conversely, every vertex $v\in V(T_i)$, $i\in \{1,\dots ,k\}$, is a vertex in *T*. Therefore, we have $V(T)=\bigcup _{i=1}^k V(T_i)$.
(iii) *T* exists if and only if the sets *L*(*T*(*x*)) and *L*(*T*(*y*)) for all $x,y\in \bigcup _{i=1}^k V(T_k)$ are either comparable by set inclusion or disjoint, i.e., $L(T(x))\cap L(T(y))\in \{L(T(x)),L(T(y)),\emptyset \}$. Thus, $x \prec _T y$ if and only if $L(T_i(x))=L(T(x)) \subsetneq L(T_j(y))=L(T(y))$ for the appropriate choices of $i,j\in \{1,\dots , k\}$.

**Fig. 1:**
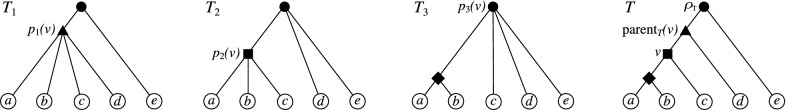
The three trees $T_1$, $T_2$, and $T_3$ with common leaf set $L=\{a,b,c,d,e\}$ have the (unique) common refinement *T*. Here, $J(\rho )=\{1,2,3\}$ and thus, ${\bar{J}}(\rho ) = \emptyset$. The different symbols for vertices indicate which vertex *u* in the $T_i$s corresponds to which vertex *u* in *T*. Consider the vertex *v* highlighted as $\blacksquare$. The corresponding vertices $p_i(v)$ are shown in the respective trees $T_i$. Here, $p_2(v)=v$ while the vertices $p_1(v)$ and $p_3(v)$ in $T_1$ and $T_3$ correspond to ${\text {parent}}_T(v)$ and $\rho$, respectively. Consequently, $J(v) = \{2\}$ and ${\bar{J}}(v)=\{1,3\}$. We have $p_2(v)=v\prec _T{\text {parent}}_T(v) = p_1(v)\prec _T p_3(b)$, according to Obs. 3. In this example, only the last case in Obs. 4 for *v* is satisfied, namely ${\text {parent}}_T(v)=p_1(v)$. Moreover, $A(v) = \{v\} \cup \{{\text {parent}}_{T_2}(v)=\rho \} \cup \{p_1(v),p_3(v)\} = \{v,\rho , {\text {parent}}_T(v)\}$

Observation (iii) makes it possible to access the ancestor order $\prec _T$ on *V*(*T*) without knowing the common refinement *T* explicitly. Many of the upcoming definitions are illustrated in Fig. 1.

We introduce, for each $v\in V(T)$, the index set $J(v)=\{i\mid L(T_i(v))=L(T(v))\}$ of the trees that contain vertex *v*. We have $J(v)\ne \emptyset$ for all $v\in V(T)$. For simplicity, we write ${\bar{J}}(v){:}{=}\{1,\dots ,k\}\setminus J(v)$ for the indices of all other trees. Hence, ${\bar{J}}(v)=\emptyset$ if and only if $L(T(v))\in {\mathcal {H}}(T_i)$ for all $i\in \{1,\dots ,k\}$. In particular, therefore, ${\bar{J}}(v)=\emptyset$ whenever $v\in L$ or $v=\rho$.

*Let us assume until further notice that a common refinement exists and let* $T=(V,E)$ *be the unique least resolved common refinement of* $T_1$, $T_2$, ..., $T_k$ *on a common leaf set.* Due to Lemma 1, *T* is uniquely determined by the parent function ${\text {parent}}_T$. The key ingredient in our construction are the following vertices in $T_i$:

1 $$\begin{aligned} p_i(v){:}{=}{\text {lca}}_{T_i}(L(T(v)),\qquad i\in \{1,\dots ,k\},\ v\in V(T) \end{aligned}$$

By assumption, we have $L(T(v))\subseteq L(T_i)$ and thus $p_i(v)$ is well-defined. As immediate consequence of the definition in Eq. (1), we have

#### Observation 3

For all $v\in V(T)$ and all $i\in \{1,\dots ,k\}$ it holds that $p_i(v)=v$ iff $v\in V(T_i)$ iff $i\in J(v)$. If $i\notin J(v)$, then $v\prec _T p_{i}(v)$ and therefore ${\text {parent}}_T(v)\preceq _T p_i(v)$.

Now assume that ${\text {parent}}_T(v)$ exists in *T*, i.e., $v\ne \rho$. By Observation (ii), $v\in V(T)$ implies $v\in V(T_i)$ for some $i\in \{1,\dots ,k\}$. In this case, ${\text {parent}}_T(v)$ must be the unique $\preceq _{T_i}$-minimal vertex $u_i\in V(T_i)$ that satisfies $L(T(v))\subsetneq L(T_i(u_i))$ because ${\mathcal {H}}(T_i)\subseteq {\mathcal {H}}(T)$. In other words, $p_i({\text {parent}}_T(v)) = u_i = {\text {parent}}_{T_i}(v)$. Hence, we have

#### Observation 4

For all $v\in V\setminus \{\rho \}$ it holds that ${\text {parent}}_T(v)={\text {parent}}_{T_i}(v)$ for some $i\in J(v)$ or ${\text {parent}}_T(v)=p_{j}(v)$ for some $j\in {\bar{J}}(v)$.

Note that in general also both cases in Obs. 4 are possible. Consider the set of vertices $A(v){:}{=}\{v\}\cup \{{\text {parent}}_{T_i}(v)\mid i\in J(v)\}\cup \{p_i(v)\mid i\in {\bar{J}}(v)\}$. By construction and Obs. 4, we have $v\preceq _T x$ for all $x\in A(v)$. Since all ancestors of a vertex in a tree are mutually comparable w.r.t. the ancestor order, we have

#### Observation 5

All $x,y\in A(v)$ are pairwise comparable w.r.t. $\preceq _T$.

Taken together, Observations 3-5 imply that the parent map of *T* can be expressed in the following form:

2 $$\begin{aligned} {\text {parent}}_T(v) = \min \left( \min _{i\in J(v)} {\text {parent}}_{T_i}(v),\ \min _{i\in {\bar{J}}(v)} p_i(v) \right) \end{aligned}$$

where the minimum is taken w.r.t. the ancestor order $\preceq _T$ on *T*. Since the root $\rho _i$ of each $T_i$ coincides with the root $\rho$ of *T*, *v* is the root of *T* iff ${\text {parent}}_{T_i}(v)=\varnothing$ is undefined for one and thus for all *i*. In this case, we set ${\text {parent}}_T(v)=\varnothing$.

With this in hand, we show how to compute the maps $p_i$ for $u{:}{=}{\text {parent}}_T(v)$ for all $i\in \{1,\dots ,k\}$. To this end, we distinguish three cases. (1) If $u\in V(T_i)$, we have $p_i(u)=u$ by definition. (2) If $u\notin V(T_i)$, then we have to identify the $\preceq _T$-minimal vertex $w\in V(T_i)$ with $u\prec _T w$. If $v\in V(T_i)$, then $p_i(u)=w={\text {parent}}_{T_i}(v)$. In the remaining case, $i\in {\bar{J}}(v)$, we already know that $p_i(v)$ is the $\preceq _{T_i}$-minimal ancestor of *v*. Thus, we have either $p_i(v)=u={\text {parent}}_T(v)$, i.e., a sub-case of (1), or (3) $u\preceq _T p_i(v)$ whenever $v\notin V(T_i)$ and $u\notin V(T_i)$. In this case, the definition of $p_i$ implies $p_i(u)=p_i(v)$. Summarizing the three cases yields the following recursion:

3 $$\begin{aligned} p_i(u) = {\left\{ \begin{array}{ll} u &{} \text { if } i\in J(u) \\ {\text {parent}}_{T_i}(v) &{} \text { if } i\in J(v) \\ p_i(v) &{} \text { if } i\in {\bar{J}}(u) \text { and } i \in {\bar{J}}(v) \end{array}\right. } \end{aligned}$$

Note, although the cases in Eq. (3) are not exclusive (since $J(v)\cap J(u)\ne \emptyset$ is possible), they are not in conflict. To see this, observe that if $i\in J(u)$ and $i\in J(v)$, then $u={\text {parent}}_{T_i}(v)$ as a consequence of the definition of *u*.

Initializing $i\in J(v)$ for all *i* and all leaves *v*, we can compute *J*(*u*) for $u={\text {parent}}_T(v)$ as a by-product by the minimum computation in Eq. (2) by simply keeping track of the equalities encountered since both ${\text {parent}}_{T_i}(v)$ and $p_i(v)$ are vertices in $T_i$. More precisely, each time a strictly $\preceq _T$-smaller vertex $u'$, i.e., a proper set inclusion, is encountered in Eq. (2), the current list of equalities is discarded and re-initialized as $\{i\}$, where *i* is the index of the tree $T_i$ in which the new minimum $u'$ was found. The indices of the trees $T_j$ with $u'\in V(T_j)$ are then appended.

It remains to ensure that the vertices are processed in the correct order. To this end, we use a queue ${\mathcal {Q}}$, which is initialized by enqueueing the leaf set. Upon dequeueing *v*, its parent *u* and the values $p_i(u)$ are computed. Except for the leaves, every vertex $u\in V(T)$ appears as parent of some $v\in V(T)$. On the other hand, *u* may appear multiple times as parent. Thus we enqueue *u* in ${\mathcal {Q}}$ only if the same vertex has not been enqueued already in a previous step. We emphasize that it is not sufficient to check whether $u\in {\mathcal {Q}}$ since *u* may have already been dequeued from ${\mathcal {Q}}$ before re-appearance as a parent. We therefore keep track of all vertices that have ever been enqueued in a set *V*. To see that this is indeed necessary, consider a tree $T_i=(a,(b,c)v_1)v_2$ and an initial queue ${\mathcal {Q}}=(a,b,c)$. Without the auxiliary set *V*, we obtain ${\mathcal {Q}}=(b,c,v_2)$, ${\mathcal {Q}}=(c,v_2,v_1)$, ${\mathcal {Q}}=(v_2,v_1)$, ${\mathcal {Q}}=(v_1)$, ${\mathcal {Q}}=(v_2)$, *etc.*, and thus $v_2$ is enqueued twice.

An implementation of this procedure also needs to keep track of the correspondence between vertices in *V*(*T*) and the vertices of $V(T_i)$. To this end, we can associate with each $v\in V(T)$ a list of pointers to $v\in V(T_i)$ for $i\in J(v)$, and pointer from $v\in V(T_i)$ back to $v\in V(T)$. For the leaves, these are assigned upon initialization. Afterwards, they are obtained for $u={\text {parent}}_{T}(v)$ as a by-product of computing *J*(*u*), since the pointers have to be set exactly for the $i\in J(u)$. In particular, whenever the pointer for *u* found $T_i$ has already been set, we know that $u\in V$.

Summarizing the discussion so far, we have shown:

#### Proposition 6

*Suppose the trees* $T_1$, $T_2$, ..., $T_k$ *have a common refinement* *T*. *Then* ${\text {parent}}_{T}(v)$ *is correctly computed by the recursions* Eq. (2) and Eq. (3).

Next we observe that it is not necessary to explicitly compute set inclusions. As an immediate consequence of Obs. 5 and the fact that $x\ne y$ implies $L(T(x))\ne L(T(y))$ because all trees are phylogenetic by assumption, we obtain

#### Observation 7

For any two $x,y\in A(v)$, we have $x\prec _T y$ if and only if $|L(T(x))|<|L(T(y))|$.

Thus it suffices to evaluate the minimum in Eq. (2) w.r.t. to the cardinalities |*L*(*T*(*v*))|. This can be achieved in *O*(*k*) time provided the values $\ell _i(v){:}{=}|L(T_i(v))|$ are known for the input trees. Since the parent-function ${\text {parent}}_T$ unambiguously defines a tree *T*, we have

#### Corollary 8

*Suppose the trees* $T_1$, $T_2$, ..., $T_k$ *have a common refinement* *T*. *Then* *T* *can be computed in* *O*(*k*|*L*|) *time*.

#### *Proof*

For each input tree $T_i$, $\ell _i(v)$ can be computed as

4 $$\begin{aligned} \ell _i(v)= {\left\{ \begin{array}{ll} 1 &{} \text {if } v\in L, \text { and} \\ \ell _i(v)=\displaystyle \sum _{u\in {\text {child}}_{T_i}(v)}\ell _i(u) &{} \text {otherwise.} \end{array}\right. } \end{aligned}$$

Since the total number of terms appearing for the inner vertices of *T* equals the number of edges of $T_i$, the total effort for $T_i$ is bounded by *O*(|*L*|). The total number of vertices *u* computed as ${\text {parent}}_{T}(v)$ equals the number of edges of *T*, and thus is also bounded by *O*(*L*). Since the tree *T*, as well as the *k* trees $T_i$, have *O*(|*L*|) vertices, we require *O*(*k*|*L*|) pointers from the vertices in *T* to their corresponding vertices in the $T_i$ and *vice versa*. By initializing the pointers for all $v\in V(T_i)$ as “not set”, it can be checked in constant time whether *u* that was found in $T_i$ is already contained in the set *V*, since this is the case if and only if its pointer has already been set. Evaluation of Eq. (2) requires *O*(*k*) comparisons, each of which can be performed in constant time by virtue of Obs. 7. The computation of $p_i(u)$ and *J*(*u*) as well as the update of the correspondence table between vertices in *T* and $T_i$, $1\le i\le k$ requires *O*(*k*) operations for each $v\in V(T)$. Thus *T* can be computed in *O*(*k*|*L*|) time. $\square$


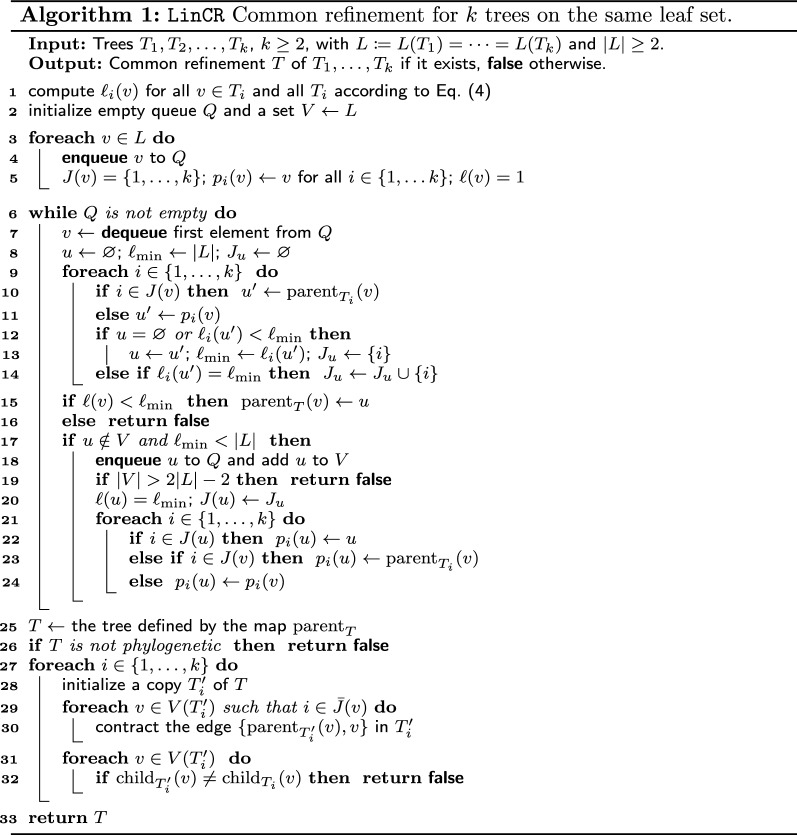


So far, we have assumed that a common refinement exists. By a slight abuse of notation, we also use the function ${\text {parent}}_T$ if the refinement *T* does not exist. In this case, we define ${\text {parent}}_T$ on the union of the $V(T_i)$ recursively by Eqs. (2) and (3). Alg. 1 summarizes the procedure based on the leaf set cardinalities for the general case. If no common refinement *T* exists, then either ${\text {parent}}_T$ does not specify a tree, or the tree *T* defined by ${\text {parent}}_T$ is not a common refinement of $T_1$, $T_2$, ..., $T_k$. The following result shows that we can always efficiently compute ${\text {parent}}_T$ and check whether it specifies a common refinement of the input trees.

#### Theorem 9

LinCR (*Alg. 1) decides in* *O*(*k*|*L*|) *time whether a common refinement of trees* $T_1$, $T_2$, ..., $T_k$ *on the same leaf set* *L* *exists and, in the affirmative case, returns the tree* *T* *corresponding to* ${\mathcal {H}}(T)={\mathcal {H}}(T_1)\cup {\mathcal {H}}(T_2)\cup \dots \cup {\mathcal {H}}(T_k)$.

#### *Proof*

We construct ${\text {parent}}_{T}$ in Lines 1–24 as described in the proof of Cor. 8. In particular, we determine $u{:}{=}{\text {parent}}_T(v)$ by virtue of the smallest $\ell _i(u)$. Hence, we can process each enqueued vertex *v* in *O*(*k*). Moreover, if a common refinement *T* exists, then Cor. 8 guarantees that we obtain this tree in Line 25.

A tree on |*L*| leaves has at most $|L|-1$ inner vertices with equality holding for binary trees. Therefore, the set *V* of distinct vertices encountered in Alg. 1, can contain at most $2|L|-2$ vertices (note that by construction the root does not enter *V*). If this condition is violated, no common refinement exists and we can terminate with a negative answer (cf. Line 19). This ensures that ${\text {parent}}_{T}$ is constructed in *O*(*k*|*L*|) time. We continue by showing that, unless the algorithm exits in Line 16 or 19, ${\text {parent}}_{T}$ in Line 25 always defines a tree *T*. To see this, consider the graph *G* with vertex set $V\cup \{\rho \}$ where $\rho$ is the root vertex which is contained in each $T_i$ and an edge $\{u,v\}$ if and only if ${\text {parent}}_{T}(v)=u$ or ${\text {parent}}_{T}(u)=v$. Checking whether $\ell (v)<\ell _{\min }(=\ell (u))$ in Line 15 ensures that *G* does not contain cycles and that ${\text {parent}}_{T}(v)=u$ *and* ${\text {parent}}_{T}(u)=v$ is not possible. Moreover, every vertex $v\in V$ is enqueued to ${\mathcal {Q}}$ and receives a parent *u* such that $\ell (v)<\ell (u)$. Unless $u=\rho$, *u* in turn receives a parent $u'$ with $\ell (u)<\ell (u')$. Since *V* is finite $v,u,u',...$ are pairwise distinct as a consequence of the cardinality condition, and we conclude that eventually $\rho$ is reached, i.e., a path to $\rho$ exists for all $v\in V$. It follows that *G* is connected, acyclic, and simple, and thus a tree (with root $\rho$).

It remains to check whether *T* is phylogenetic and displays $T_i$ for all $i\in \{1,\dots ,k\}$. Checking whether *T* is phylogenetic in Line 26 can be done in *O*(|*L*|) in a top-down traversal that exits as soon as it encounters a vertex with a single child. To check whether *T* displays a tree $T_i$, we contract (in a copy of *T*) in a top-down traversal all edges *uv* with $v\in {\text {child}}_T(u)$ for which $u\notin V(T_i)$, i.e., for which $i\notin J(v)$. Since the root of *T* and leaves of *T* are in $T_i$, this results in a rooted tree $T_i'$ with $V(T_i)=V(T_i')$ if *T* is indeed the common refinement of all trees. The contraction of an edge *uv* can be performed in $O({\text {child}}_T(v)|)$, hence in total time $O(|E(T_i)|)=O(|L|)$. Finally, we can check in *O*(|*L*|) time whether the known correspondence between the vertices of $T_i$ and $T_i'$ is an isomorphism. To this end, it suffices to traverse $T_i$ and to check that ${\text {child}}_{T_i}(v)={\text {child}}_{T'_i}(v)$ for all $v\in V(T_i)$ (cf. Lines 31–32) using the pointers of *v* and all elements in ${\text {child}}_{T_i}(v)$ to the corresponding vertices in *T*. Note that, in general, the pointer from a vertex *v* in $T_i$ to a vertex in $T'_i$ may not be set, in which case $v\notin V(T'_i)$ and thus, we can terminate with a negative answer. The total effort thus is bounded by *O*(*k*|*L*|).

If *T* on *L* is a phylogenetic tree displaying all trees $T_1$, $T_2$, ..., $T_k$, then it is a common refinement of these trees. Since every vertex $v\in V(T)$ is also contained in some $T_i$, i.e., $L(T(v))=L(T_i(v))$, we have ${\mathcal {H}}(T)={\mathcal {H}}(T_1)\cup {\mathcal {H}}(T_2)\cup \dots \cup {\mathcal {H}}(T_k)$. $\square$

## Computational results

We compare the running times for (a) BUILD [3], (b) BuildST [7], (c) ${\texttt {Merge\_Trees}}$ [18], (c’) ${\texttt {Loose\_Cons\_Tree}}$ [18], and (d) LinCR (Alg. 1). To this end, we implemented all of these algorithms in Python as part of the tralda library. We note that BUILD operates on a set of triples extracted from the input trees rather than the trees themselves. We use the union of the minimum cardinality sets of representative triples of every $T_i$ appearing in the proof of Thm. 2.8 in [23]. Therefore, we have $R\in O(k|L|^2)$ [24, Thm. 6.4] and BUILD runs in $O(k|L|^3)$ time. In the case of ${\texttt {Merge\_Trees}}$, we implemented a variant that starts with $T=T_1$ and then iteratively merges the clusters of the tress $T_i$, $2\le i\le k$, into *T*. ${\texttt {Merge\_Trees}}$ assumes that the input trees are compatible, which is guaranteed in our benchmarking data set. In practice, however, this condition may be violated, in which case the behavior of ${\texttt {Merge\_Trees}}$ is undefined. We therefore also implemented an *O*(*k*|*L*|) algorithm for constructing the *loose consensus tree* for a set of trees $T_1$, $T_2$, ..., $T_k$ on the same leaf set, ${\texttt {Loose\_Cons\_Tree}}$, following [18]. The loose consensus comprises all clusters that occur in at least one tree $T_i$, $1\le i\le k$ and that are compatible with all other clusters of the input trees (see [25–27] and the references therein). The loose consensus tree by definition coincides with the common refinement whenever the latter exists. ${\texttt {Loose\_Cons\_Tree}}$ uses ${\texttt {Merge\_Trees}}$ as a subroutine but ensures compatibility in each step by first deleting incompatible clusters in one of the trees. This is implemented as the deletion of the corresponding inner vertex *v* followed by reconnecting the children of *v* to the parent of *v*. The input trees are compatible if and only if no deletion is necessary. The existence of a common refinement can therefore by checked by keeping track of the number of deletions. However, the subroutine that processes trees to remove incompatible clusters significantly adds to the running time of the $\texttt {Loose\_Cons\_Tree}$ algorithm. The linear-time algorithms require *O*(*k*|*L*|) space.

We simulate test instances as follows: First, a random tree $T^*$ is generated recursively by starting from a single vertex (which becomes the root) and stepwise attaching new leaves to a randomly chosen vertex *v* until the desired number of leaves |*L*| is reached. In each step, we add two children to *v* if *v* is currently a leaf, and only a single new leaf otherwise. This way, the number of leaves increases by exactly one in each step and the resulting tree $T^*$ is phylogenetic (but in general not binary). From $T^*$, we obtain $k\in \{2,8,32\}$ trees $T_1$, $T_2$,..., $T_k$ by random contraction of inner edges in (a copy of) $T^*$. Each edge is considered for contraction independently with a probability $p\in \{0.1,0.5,0.9\}$. Therefore, $T^*$ is a refinement of $T_i$ for all $1\le i\le k$, i.e., a common refinement exists by construction. However, in general we have ${\mathcal {H}}(T^*)\ne \bigcup _{i=1}^k {\mathcal {H}}(T_i)$, i.e., $T^*$ is not necessarily the minimal common refinement of the $T_i$. The trees $T_1$, $T_2$, ..., $T_k$ constructed in this manner serve as input for all algorithms.

**Fig. 2:**
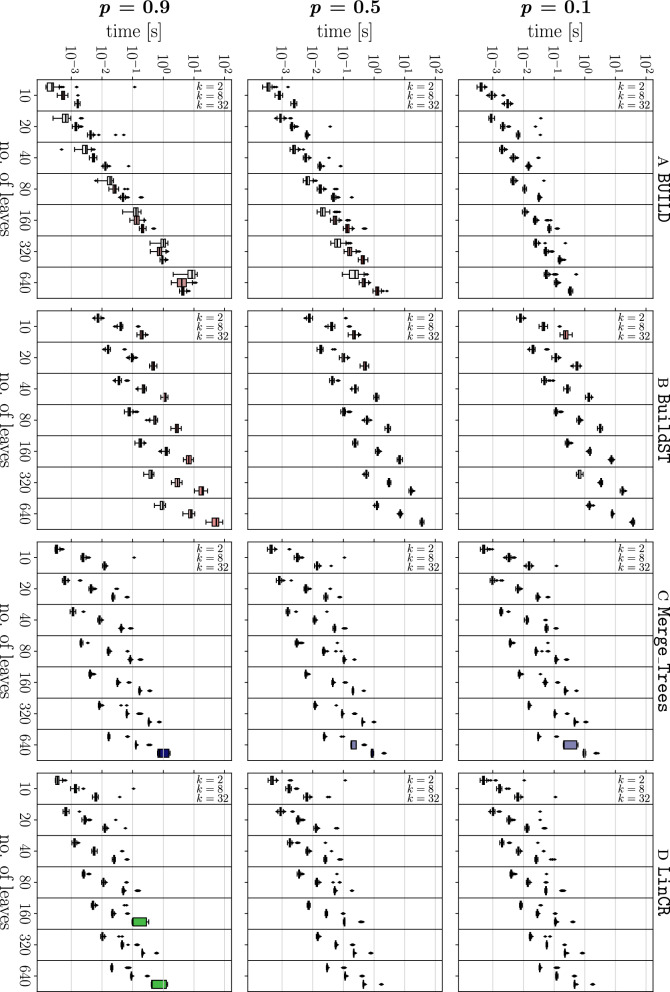
Running time comparison of the algorithms for the construction of a common refinement of *k* input trees on leaf set *L*. The subplots of each row show boxplots for the running time for different numbers of leaves |*L*| (indicated on the x-axis) and different values of $k\in \{2, 8, 32\}$ (indicated in the leftmost column of each subplot). In each row, a different probability $p\in \{0.1, 0.5, 0.9\}$ for edge contraction was used to produce the *k* input trees. Per combination of the parameters |*L*|, *k*, and *p*, 100 instances were simulated to which all four algorithm were applied

**Fig. 3:**
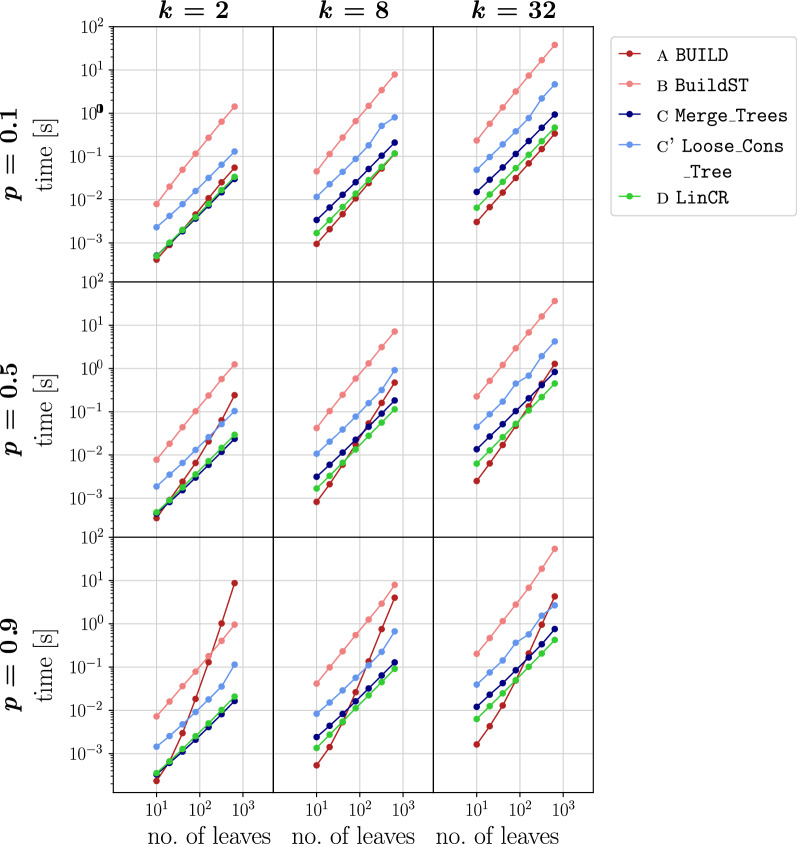
Running time comparison of the algorithms for the construction of a common refinement of *k* input trees on leaf set *L*. Per combination of the parameters |*L*| (indicated on the horizontal axis), *k* (columns), and *p* (rows), 100 instances were simulated and median values are shown for all algorithms. In each row, a different probability $p\in \{0.1, 0.5, 0.9\}$ for edge contraction was used to produce the *k* input trees

The running time comparisons were performed using tralda on an off-the-shelf laptop (Intel$^{\textregistered }$ Core$^{\mathrm{TM}}$ i7-4702MQ processor, 16 GB RAM, Ubuntu 20.04, Python 3.7). The time required to compute a least resolved common refinement of the input trees is included in the respective total running time shown in Figs. 2 and 3 . The empirical performance data are consistent with the theoretical result that LinCR scales linearly in *k*|*L*|. In particular, the median running times scale linearly with |*L*|, as shown by the slopes of $\approx 1$ in the log/log plot for the running times of LinCR in Fig. 3.

In accordance with the theoretical complexity of $O(k|L| \log ^2(k|L|))$ for the common refinement problem, the performance curve of BuildST is almost parallel to that of LinCR; however, its computation cost is higher by almost two orders of magnitude. Our implementation of BuildST uses an algorithm for dynamic graph connectivity often referred to as HDT data structure [28] as originally described in [7]. While we do not expect BuildST to become competitive with the other algorithms, we note that a recent experimental study showed that a simplified version of the HDT data structure (with a slightly worse asymptotic bound) outperforms the full version in practice [29]. For both LinCR and BuildST, the contraction probability *p* appears to have little effect on the running time. In both cases, a larger value of *p* (i.e., a lower average resolution of the input trees) leads to a moderate decrease of the running time.

In contrast, the resolution of the input trees has a large impact on the efficiency of BUILD. It also scales nearly linearly when the resolution of the individual input trees $T_i$ is comparably high (and even terminates faster than LinCR up until a few hundred leaves, cf. top-right panel), whereas its performance drops drastically with increasing *p*, i.e., for poorly resolved input trees. The reason for this is most likely the cardinality of a minimal triple set that represents the set of input trees. For binary trees, the cardinality of the triple set of $T_i$ equals the number of inner edges [23], i.e., there are *O*(|*L*|) triples. For very poorly resolved trees, on the other hand, $O(|L|^2)$ triples are required [24], matching the differences of the slopes with *p* observed for BUILD in Fig. 3.

As expected, the curves of the two *O*(*k*|*L*|) algorithms $\texttt {Merge\_Trees}$ and $\texttt {Loose\_Cons\_Tree}$ are also almost parallel to that of LinCR in Fig. 3. For $k=2$, we can even observe that $\texttt {Merge\_Trees}$ is slightly faster than LinCR. However, the smaller number of necessary tree traversals in LinCR apparently becomes a noticeable advantage with an increasing number *k* of input trees. The additional tree processing steps in the more practically relevant $\texttt {Loose\_Cons\_Tree}$ algorithm, furthermore, result in a longer running time compared to our new approach.

## Concluding remarks

We developed a linear-time algorithm to compute the common refinement of trees on the same leaf set. In contrast to the “classical” supertree algorithms BUILD and BuildST, LinCR uses a bottom-up instead of a top-down strategy. This is similar to $\texttt {Loose\_Cons\_Tree}$ and its subroutine $\texttt {Merge\_Trees}$ [18], which can also be used to obtain the common refinement of trees on the same leaf set in linear time. LinCR, however, requires fewer tree traversals and is, in our opinion, simpler to implement. In contrast to $\texttt {Merge\_Trees}$, LinCR in particular does not rely on a data structure that enables linear-time tree preprocessing and constant-time last common ancestor queries for the nodes in the tree [30]. All algorithms were implemented in Python and are freely available for download from https://github.com/david-schaller/tralda as part of the tralda library. Empirical comparisons of running times show that LinCR consistently outperforms the linear-time alternatives. Only BUILD is faster for very small instances and moderate-size trees that are nearly binary.

Although it may be possible to improve Alg. 1 by a constant factor, it is asymptotically optimal, since the input size is *O*(*k*|*L*|) for *k* trees with |*L*| leaves. Furthermore, trivial solutions can be obtained in some limiting cases. For instance, if $|V(T_i)|=2|L|-1$, then $T_i$ is binary, i.e., no further refinement is possible. In this case, we can immediately use $T=T_i$ as the only viable candidate and only check that $T_j$ displays all other $T_j$. However, we cannot entirely omit Lines 1–24 in this case since we require the sets *J*(*v*) as well as the correspondence between the vertices in order to check whether *T* displays every $T_i$.

It is worth noting that the idea behind LinCR does not generalize to more general supertree problems. The main reason is that the set inclusions employed to determine $\prec _T$ do not carry over to the more general case because the inclusion order of $C_1,C_2\in {\mathcal {H}}(T)$ cannot be determined from $C_1\cap L(T_i)$ and $C_2\cap L(T_j)$ for two trees with $L(T_i),L(T_j)\subsetneq L(T)$.

Depending on the application, a negative answer to the existence of a common refinement may not be sufficient. One possibility is to resort to the loose consensus tree or possibly other notions of consensus trees, see e.g. [25, 31]. A natural alternative approach is to extract a maximum subset of consistent triples from $\bigcup _{i=1}^k r(T_i)$. This problem, however, is known to be NP-hard for arbitrary triple sets, see e.g. [32] and the references therein.

## Data Availability

Implementations of the algorithms used in this contribution are available at https://github.com/david-schaller/tralda as part of the tralda library.

## References

[1] Sanderson MJ, Purvis A, Henze C (1998). Phylogenetic supertrees: assembling the trees of life. Trends Ecol Evol.

[2] Semple C, Steel M (2000). A supertree method for rooted trees. Discr Appl Math.

[3] Aho AV, Sagiv Y, Szymanski TG, Ullman JD (1981). Inferring a tree from lowest common ancestors with an application to the optimization of relational expressions. SIAM J Comput.

[4] Semple C, Steel M (2003). Phylogenetics.

[5] Constantinescu M, Sankoff D (1995). An efficient algorithm for supertrees. J Classif.

[6] Henzinger MR, King V, Warnow T (1999). Constructing a tree from homeomorphic subtrees, with applications to computational evolutionary biology. Algorithmica.

[7] Deng Y, Fernández-Baca D (2018). Fast compatibility testing for rooted phylogenetic trees. Algorithmica.

[8] Deng Y, Fernández-Baca D (2017). An efficient algorithm for testing the compatibility of phylogenies with nested taxa. Algorithms Mol Biol..

[9] Geiß M, Chávez E, González Laffitte M, López Sánchez A, Stadler BMR, Valdivia DI, Hellmuth M, Hernández Rosales M, Stadler PF (2019). Best match graphs. J Math Biol..

[10] Schaller D, Geiß M, Chávez E, González Laffitte M, López Sánchez A, Stadler BMR, Valdivia DI, Hellmuth M, Hernández Rosales M, Stadler PF (2021). Corrigendum to “Best Match Graphs”. J Math Biol..

[11] Geiß M, Anders J, Stadler PF, Wieseke N, Hellmuth M (2018). Reconstructing gene trees from Fitch’s Xenology relation. J Math Biol..

[12] Hellmuth M, Seemann CR (2019). Alternative characterizations of Fitch’s Xenology relation. J Math Biol..

[13] Hellmuth M, Michel M, Nøjgaard N, Schaller D, Stadler PF, Stadler PF, Walter MEMT, Hernandez-Rosales M, Brigido MM (2021). Combining orthology and xenology data in a common phylogenetic tree. Advances in bioinformatics and computational biology.

[14] Warnow TJ (1994). Tree compatibility and inferring evolutionary history. J Algorithms.

[15] Gusfield D (1991). Efficient algorithms for inferring evolutionary trees. Networks.

[16] Aho AV, Hopcroft JE, Ullman JD (1974). The design and analysis of computer algorithms.

[17] Jansson J, Shen C, Sung W-K. Improved algorithms for constructing consensus trees. In: Khanna, S. (ed.) Proceedings of the 2013 Annual ACM-SIAM Symposium on Discrete Algorithms (SODA), pp. 1800–1813. Soc. Indust. Appl. Math., Philadelphia, PA 2013. 10.1137/1.9781611973105.129.

[18] Jansson J, Shen C, Sung W-K (2016). Improved algorithms for constructing consensus trees. J ACM.

[19] Hellmuth M, Schaller D, Stadler PF. Compatibility of partitions with trees, hierarchies, and split systems 2021. submitted; arXiv:2104.14146.

[20] Bryant D, Steel M (1995). Extension operations on sets of leaf-labeled trees. Adv Appl Math..

[21] Semple C (2003). Reconstructing minimal rooted trees. Discr Appl Math..

[22] Jansson J, Lemence RS, Lingas A (2012). The complexity of inferring a minimally resolved phylogenetic supertree. SIAM J Comput..

[23] Grünewald S, Steel M, Swenson MS (2007). Closure operations in phylogenetics. Math Biosci..

[24] Seemann CR, Hellmuth M (2018). The matroid structure of representative triple sets and triple-closure computation. Eur J Comb..

[25] Bremer K (1990). Combinable component consensus. Cladistics.

[26] Day WHE, McMorris FR. Axiomatic Consensus Theory in Group Choice and Bioinformatics. Society for Industrial and Applied Mathematics, Providence, RI 2003. 10.1137/1.9780898717501.

[27] Dong J, Fernández-Baca D, McMorris FR, Powers RC (2011). An axiomatic study of majority-rule (+) and associated consensus functions on hierarchies. Discr Appl Math..

[28] Holm J, de Lichtenberg K, Thorup M (2001). Poly-logarithmic deterministic fully-dynamic algorithms for connectivity, minimum spanning tree, 2-edge, and biconnectivity. J ACM.

[29] Fernández-Baca D, Liu L (2019). Tree compatibility, incomplete directed perfect phylogeny, and dynamic graph connectivity: an experimental study. Algorithms.

[30] Bender MA, Farach-Colton M, Pemmasani G, Skiena S, Sumazin P (2005). Lowest common ancestors in trees and directed acyclic graphs. J Algorithms.

[31] Bryant D, Janowitz MF, Lapointe F-J, McMorris FR, Mirkin B, Roberts FS (2003). A classification of consensus methods for phylogenetics. Bioconsensus.

[32] Byrka J, Guillemot S, Jansson J (2010). New results on optimizing rooted triplets consistency. Discr Appl Math..

